# The trajectories of depressive symptoms among working adults during the COVID-19 pandemic: a longitudinal analysis of the InHamilton COVID-19 study

**DOI:** 10.1186/s12889-021-11900-8

**Published:** 2021-10-19

**Authors:** Divya Joshi, Andrea Gonzalez, Lauren Griffith, Laura Duncan, Harriet MacMillan, Melissa Kimber, Brenda Vrkljan, James MacKillop, Marla Beauchamp, Nick Kates, Parminder Raina

**Affiliations:** 1 Department of Health Research Methods, Evidence, and Impact, McMaster University, Hamilton, Ontario Canada; 2 Labarge Centre for Mobility in Aging, McMaster University, Hamilton, Ontario Canada; 3 McMaster Institute for Research on Aging, McMaster University, Hamilton, Ontario Canada; 4 Department of Psychiatry & Behavioural Neurosciences, McMaster University, Hamilton, Ontario Canada; 5 Offord Centre for Child Studies, Hamilton, Ontario Canada; 6 Department of Pediatrics, McMaster University, Hamilton, Ontario Canada; 7 School of Rehabilitation Science, McMaster University, Hamilton, Ontario Canada

**Keywords:** Depressive symptoms, Mental health, COVID-19, Caregiving, Coping strategies, Employed adults, Growth mixture modeling

## Abstract

**Background:**

Longitudinal studies examining the impact of changes in COVID-19 pandemic-related stressors and experiences, and coping styles on the mental health trajectory of employed individuals during the lockdown are limited. The study examined the mental health trajectories of a sample of employed adults in Hamilton, Ontario during the initial lockdown and after the re-opening following the first wave in Canada. Further, this study also identified the pandemic-related stressors and coping strategies associated with changes in depressive symptoms in employed adults during the COVID-19 pandemic.

**Methods:**

The InHamilton COVID-19 longitudinal study involved 579 employees aged 22–88 years from a large public university in an urban area of Hamilton, Ontario at baseline (April 2020). Participants were followed monthly with 6 waves of data collected between April and November 2020. A growth mixture modeling approach was used to identify distinct groups of adults who followed a similar pattern of depressive symptoms over time and to describe the longitudinal change in the outcome within and among the identified sub-groups.

**Results:**

Our results showed two distinct trajectories of change with 66.2% of participants displaying low-consistent patterns of depressive symptoms, and 33.8% of participants displaying high-increasing depressive symptom patterns. COVID-19 pandemic-related experiences including health concerns, caregiving burden, and lack of access to resources were associated with worsening of the depressive symptom trajectories. Frequent use of dysfunctional coping strategies and less frequent use of emotion-focused coping strategies were associated with the high and increasing depressive symptom pattern.

**Conclusions:**

The negative mental health impacts of the COVID-19 pandemic are specific to subgroups within the population and stressors may persist and worsen over time. Providing access to evidence-informed approaches that foster adaptive coping, alleviate the depressive symptoms, and promote the mental health of working adults is critical.

## Introduction

The emergence and rapid spread of the severe acute respiratory syndrome coronavirus 2 (SARS-CoV-2) created a global health crisis that prompted governments around the world to implement a range of public health measures to control the spread of the infection [1]. The first lockdown began in most Canadian provinces, including Ontario, in mid-March 2020 and continued until June 2020. During this period, provincial and territorial governments implemented public health measures to mitigate the transmission of SARS-CoV-2. Examples of these measures include, but are not limited to, active surveillance for suspected cases, self-isolation and home quarantine; physical distancing and masking mandates; closure of schools and universities; closure of non-essential services, workplaces, and gathering places; international travel restrictions and closure of the Canada-U.S. border [1].

The COVID-19 pandemic has also negatively impacted the economy. Many individuals have experienced job insecurity as well as loss of employment and income, which has negatively impacted their mental health [2]. During the pandemic, the work environment has significantly changed, with one third of Canadian employees directed to work remotely from home [3]. For some individuals, remote work has enabled better integration of family and work responsibilities, and increased work efficiency and well-being in the short-term [4, 5]. However, for others, remote work introduced barriers to productivity, such as lack of interaction with colleagues, inadequate workspace, difficulty with internet speed, and limitations with accessing work-related information and devices. Such barriers have been described as a blurring of the work-home boundaries, that has been attributed to lower productivity and motivation, greater emotional exhaustion, and higher stress [6–9]. These negative effects may vary depending upon the level of support provided by an employer, the individual’s social network outside of work, types of coping strategies used, and demands of the home environment [6–9]. Studies have also reported that individuals with greater caregiving responsibilities and/or those who report feeling an added burden in terms of caring for others during disasters, including COVID-19, identify higher levels of stress and anxiety [10–12]. As those who are working remotely or otherwise continue to adhere to the physical distancing policies, it is important to examine the impact of the COVID-19 pandemic and related stressors on their respective mental health.

Studies that compared data before the pandemic with that collected during lockdowns have shown an increase in the prevalence of psychological distress, depressive symptoms, and post-traumatic stress disorder [13, 14]. However, research examining the trajectory of mental health of employees during the initial lockdown and re-opening phases of the COVID-19 pandemic is needed. Hence, it remains unclear as to whether the mental health of employed adults worsened as the lockdown continued and after the restrictions were gradually lifted, or whether the trends indicated stabilization or improvement in mental health. Longitudinal studies examining the impact of changes in COVID-19 pandemic-related stressors, experiences, and coping styles on the mental health trajectory during the lockdown are limited. The purpose of this study was to examine the mental health trajectories of employed adults during the initial lockdown and as the public health restrictions eased following the first wave in Hamilton, Ontario. This study also identified the pandemic-related stressors and coping strategies associated with depressive symptoms in working population during the COVID-19 pandemic.

## Methods

### Study design and participants

The InHamilton COVID-19 longitudinal study was designed to understand the impact of the COVID-19 pandemic in employed adults. A total of 3800 participants including full-time and part-time faculty and staff from McMaster University, Hamilton, Ontario, were invited to take part in the study. The study recruited 579 faculty and staff at baseline (April 2020), to be followed monthly from April to August 2020 (5 monthly questionnaires), with the exit questionnaire being administered in November 2020. Hamilton, Ontario is a city of approximately 537,000 and is part of the Greater Toronto and Hamilton Area. McMaster University is a large public university in Hamilton, with 15,900 part-time and full-time employees, and is one of city’s largest employer. Thus, the sample reflects adults working for a larger institutional employer in an urban-suburban catchment area. Participants were recruited via an e-mail sent by the university’s Human Resources department to all faculty and staff, inviting them to complete a web-based questionnaire. Participants were provided with information about the purpose of the study before obtaining their consent. At baseline, information was collected about demographic factors, COVID-19 related stressors and experiences, COVID-19 symptoms, social distancing behaviours, lifestyle behaviours, physical health conditions, impact of the COVID-19 pandemic on children, family relationships, partner conflicts, coping strategies, and mental health consequences of the pandemic. This study was approved by the Hamilton Integrated Research Ethics Board (Number 8024).

### Study measures

#### Depressive symptoms

Screening for depressive symptoms was completed using the 10-item Center for Epidemiologic Studies Short Depression Scale (CESD-10), which assesses depressive symptoms in the past week [15]. This scale includes eight items on depressed affect and two items on positive affect. Each item includes four response categories ranging from 0 to 3: rarely or never (less than 1 day), some of the time (1–2 days), occasionally (3–4 days), and all of the time (5–7 days). Scores for each participant were summed after reversing the positive affect items and can range between 0 and 30 with higher scores indicating greater number of depressive symptoms. A score of 10 or higher is identified as clinically significant depressive symptoms [15]. The CESD-10 is shown to be reliable and valid in assessing depressive symptoms in adults, with internal consistency of 0.86, test-retest reliability of 0.85, convergent validity of 0.91, and divergent of 0.89 [16–19]. The CESD-10 was administered at baseline and at each follow-up time point.

#### COVID-19 experiences scale

Data on COVID-19 pandemic-related experiences and stressors were measured using a self-report questionnaire [20] where participants were asked to indicate their experiences during the past month. This questionnaire was administered at baseline and at each follow-up time point. Health-related stressors were identified by asking participants to indicate whether they were ill or if someone close to them was ill, hospitalized, or had died within the past month for COVID-19 or non-COVID-19 related reasons. Difficulties with accessing resources was identified by asking participants to indicate whether they had experienced loss of income, difficulties in accessing necessary supplies or food, and were unable to get usual healthcare and prescription medications and treatments. Caregiving experience during the pandemic was identified by asking participants to indicate whether they had spent increased time caring for young and/or school-aged children, interacting with adolescents, and caregiving for older adults, and whether they were unable to care for people who require assistance due to health condition or limitation [20, 21]. Health-related stressors, difficulties with accessing resources, and caregiving experiences were grouped as ‘yes’ if the participant indicated at least one experience in the specific category or ‘no’ if the participant did not indicate any of the experiences in the specific category.

#### Coping strategies

The Brief COPE, a 28-item self-reported questionnaire that examines [22]. Each item is scored on a 4-point Likert scale ranging from “I have not been doing this a lot” (0 points) to “I have been doing this a lot” (3 points). The 28 items are combined into 14 subscales: Self-distraction, active coping, denial, substance use, emotional support, use of instrumental support, behavioural disengagement, venting, positive reframing, planning, humour, acceptance, religion, and self-blame. Subscales were grouped into three coping strategies: 1) ‘problem-focused’ strategy was based on the sum of scores on the active coping, planning, and use of instrumental support subscales; 2) ‘emotion-focused’ strategy was based on the sum of scores on the positive reframing, acceptance, humour, religion, and emotional support subscales; and 3) ‘dysfunctional coping’ strategy was based on the sum of scores on the self-distraction, denial, venting, substance use, behavioural disengagement, and self-blame subscales. The score for each subscale can range from 0 to 6 with higher score indicating more frequent use of the coping style. The Brief COPE has been validated in a community sample impacted by a natural disaster with internal consistency between 0.50 and 0.90 for the individual subscales [23].

### Statistical analysis

Descriptive statistics were reported at each time point. The time was coded as wave 1 (baseline) to wave 6 (exit questionnaire). The latent growth mixture model was used to estimate group-based trajectories of depressive symptoms based on the CESD-10 score [24, 25]. The growth mixture modeling approach is used to identify unobserved sub-populations of individuals who follow a similar pattern of the outcome over time and describe the longitudinal change in the outcome within and among the unobserved sub-populations. The model allows for specification of different types of terms such as linear, quadratic, and cubic terms, to characterize the pattern over time. We used the censored normal distribution (CNORM) as depressive symptom score was modeled as a continuous variable and because depressive symptom scores cluster at their minimum values. The objective of model selection was to identify and describe the distinctive patterns of the data in the most useful and parsimonious manner [25]. Model selection involved the iterative estimation of the number of trajectory groups and the shape and order of each trajectory group. Statistical criteria for selecting the best fitting model was based on the Akaike’s Information Criterion (AIC) and Bayesian Information Criterion (BIC) with smaller values suggestive of a good fitting model. Statistically non-significant cubic and quadratic terms were excluded from the model. In some cases, the BIC value may continue to decrease as more trajectory groups are added to the model, thus, a balance between model fit statistics and usefulness of the results were considered. Missing longitudinal data were handled in the PROC TRAJ procedure under the missing at random assumption, where patterns with missing data can borrow parameter information from patterns with more data points through the creation of a latent variable [26]. COVID-19 pandemic-related experiences were included as time-varying covariates. The following covariates assessed at baseline – coping strategies, age (less than 50, 50 and over), sex (male, female), education status (below bachelor’s degree, bachelor’s degree and above), partner status (married or living with a partner in a common-law relationship, singe), type of dwelling (house, apartment), total number of COVID-19 symptoms, and total number of chronic health conditions - were included as predictors of group trajectory membership. Total number of COVID-19 symptoms and health conditions were assessed using a self-reported questionnaire and were included in the analysis as continuous variables. Odds ratio (OR), 95% confidence intervals (95% CI), and *p*-values were reported, and statistical analysis was conducted using PROC TRAJ Procedure in SAS software version 9.4.

## Results

**Fig. 1:**
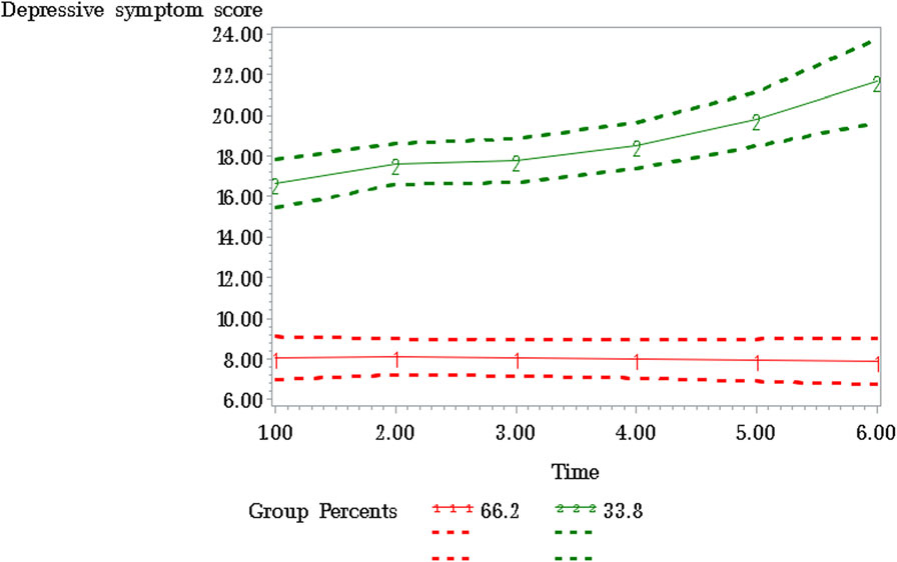
Depressive symptom trajectories (solid lines), with 95% confidence intervals (dashed lines) during the COVID-19 pandemic. Note: The dotted line indicates the threshold value (10) for clinically significant depressive symptoms

Of the 579 participants recruited at baseline (wave 1), 331 participants provided information at wave two, 300 at wave three, 260 at wave four, 200 at wave five, and 131 participants who completed the exit questionnaire (wave 6). The majority of the sample was 50 years and older (56.6%), female (79.4%), had at least a bachelor’s education (72.0%), married or living with a partner in common-law (71.7%), and resided in a house rather than an apartment (82.9%). The growth-based trajectory model specifying two trajectory groups, one with a linear term and the other with a quadratic term, was identified as the best-fitting model with a BIC value of − 4863.55 and AIC value of − 4801.49. Based on the trajectories in Fig. 1, the distinct depressive symptom trajectory groups were labeled *low and consistent,* and *high and increasing.* The low and consistent depressive symptom trajectory group was comprised of 66.2% of participants, and their depressive symptoms remained consistently low during the follow-up period and below the threshold of 10 for clinical significance. The high and increasing depressive symptom trajectory group, comprising 33.8% of participants, which reflected a quadratic pattern with depressive symptoms increasing from timepoint 1 to timepoint 6.

The characteristics of participants in the two depressive symptoms trajectory groups are presented in Table 1. When compared to the low and consistent depressive symptoms trajectory group, the high and increasing depressive symptoms trajectory group had a larger proportion of individuals aged 50 years and under (65.33% vs. 50.98%, *p*-value: 0.0037), females (85.52% vs. 76.90%, *p*-value: 0.0339), had lower educational attainment (i.e., below a bachelor’s degree) (31.33% vs. 22.55%, *p*-value: 0.0430), not married or not living with a partner in common-law (38.51% vs. 22.67%, p-value: 0.0004), resided in an apartment (25.33% vs. 13.40%, p-value: 0.0016), and had clinically significant depressive symptoms (94.00% vs. 24.51%, p-value: <.0001). Table 2 shows the depression score and COVID-19 stressors and experiences over the six time points. Overall, the average depression score at baseline was 9.63 (standard deviation (SD): 6.15) and remained relatively consistent throughout the follow-up period. At baseline, 21.93% of participants reported health-related concerns, 30.33% reported difficulties with accessing resources, and 33.20% reported spending more time in caregiving responsibilities or were unable to provide care for family members due to pandemic restrictions.

**Table 1:** Distribution of demographic, health, and coping factors for the overall sample and between the two latent trajectory groups of depressive symptoms^a^

Variables	Overall sample	Depression Trajectories		***p***-value
		Low and consistent	High and increasing	
Age, n (%)				0.0037
< 50 years	297 (56.57)	156 (50.98)	98 (65.33)	
≥ 50 years	228 (43.43)	150 (49.02)	52 (34.67)	
Sex, n (%)				0.0339
Male	106 (20.58)	70 (23.10)	21 (14.48)	
Female	409 (79.42)	233 (76.90)	124 (85.52)	
Education, n (%)				0.0430
Bachelor’s degree or above	378 (72.00)	237 (77.45)	103 (68.67)	
Below bachelor’s degree	147 (28.00)	69 (22.55)	47 (31.33)	
Partner status, n (%)				0.0004
Married or living with a partner in common-law	370 (71.71)	232 (77.33)	91 (61.49)	
Single	146 (28.29)	68 (22.67)	57 (38.51)	
Type of dwelling, n (%)				0.0016
House	432 (82.92)	265 (86.60)	112 (74.67)	
Apartment	89 (17.08)	41 (13.40)	38 (25.33)	
Number of COVID-19 related symptoms, mean (SD)	4.17 (3.24)	3.60 (2.78)	5.43 (3.68)	<.0001
Number of chronic conditions, mean (SD)	1.71 (1.80)	1.75 (1.63)	2.49 (2.03)	<.0001
Coping strategy				
Problem-focused, mean (SD)	7.29 (3.87)	7.35 (3.79)	7.10 (4.03)	0.5123
Emotion-focused, mean (SD)	12.99 (4.91)	13.51 (5.01)	11.83 (4.45)	0.0006
Dysfunctional, mean (SD)	7.27 (4.66)	5.59 (3.33)	10.78 (5.08)	<.0001
Clinically significant depressive symptoms, n (%)	225 (46.11)	75 (24.51)	141 (94.00)	<.0001

^a^Participants in the low and consistent trajectory group displayed consistently low levels of depressive symptoms, while participants in the high and increasing trajectory group displayed high and accelerated increase in their depressive symptoms over time. P-value is for the comparison of characteristics between the two trajectory groups

**Table 2:** Change in depression score and COVID-19 stressors and experiences for all participants over the follow-up period

	Wave 1 ***n*** = 488	Wave 2 ***n*** = 318	Wave 3 ***n*** = 294	Wave 4 ***n*** = 253	Wave 5 ***n*** = 194	Wave 6 ***n*** = 129
**Variables**						
**Depression score, mean (SD)**	9.63 (6.15)	9.58 (6.29)	9.18 (6.13)	9.04 (6.61)	9.05 (6.84)	9.50 (6.92)
**Health-related stressors, n (%)**	107 (21.93)	69 (21.70)	62 (21.09)	50 (19.76)	48 (24.74)	38 (29.46)
**Difficulty accessing resources, n (%)**	148 (30.33)	110 (34.59)	68 (23.13)	44 (17.39)	32 (16.49)	26 (20.16)
**Caregiving responsibilities, n (%)**	162 (33.20)	135 (42.45)	114 (38.78)	77 (30.43)	71 (36.60)	36 (27.91)

^a^The sample size reported for each wave is the number of participants with data available for depressive symptoms variable

Results identifying predictors of trajectory group membership (i.e., high-increasing vs. low-consistent) and time-varying covariates associated with depressive symptoms are presented in Table 3. In multivariable analysis, emotion-focused and dysfunctional coping strategies were significant predictors of trajectory group membership. Participants who were more likely to use a dysfunctional coping strategy had 1.36 (95% CI: 1.26, 1.47) times higher odds of being in the high-increasing depressive symptoms trajectory group whereas those who indicated greater use of an emotion-focused coping strategy had 0.85 (95% CI: 0.79, 0.92) times lower odds of being in this group. All three time-varying covariates (i.e., difficulties in accessing resources, caregiving stressors, and health-related stressors) significantly influenced the trajectory level of one or both groups. At a given trajectory time point, presence of difficulties in accessing resources and caregiving stressors were associated with worsening of the depressive symptoms of both the low-consistent and high-increasing trajectory groups, and presence of health-related stressors was associated with worsening of the depressive symptoms of the low-consistent trajectory group (Table 3).

**Table 3:** Predictors of trajectory group membership and time-varying covariates associated with depressive symptom trajectory groups during the COVID-19 pandemic

Predictors of group membership	OR^**a**^	95% CI	***p***-value
**High-increasing vs low-consistent trajectory group**			
Age (Ref. = ≥50)			
< 50	1.68	(0.88, 3.21)	0.1112
Sex (Ref. = Male)			
Female	1.57	(0.74, 3.30)	0.2377
Education status (Ref. = Bachelor’s education or above)			
Below bachelor’s education	1.73	(0.89, 3.38)	0.1031
Partner status (Ref. = Married or living with a partner in common-law)			
Single	1.34	(0.67, 2.65)	0.3994
Dwelling type (Ref. = House)			
Apartment	2.08	(0.97, 4.46)	0.0614
Number of COVID-19 related symptoms	1.11	(1.00, 1.22)	0.0573
Number of chronic conditions	1.14	(0.95, 1.36)	0.1631
Coping strategy			
Problem-focused	1.01	(0.92, 1.11)	0.8369
Emotion-focused	0.85	(0.79, 0.92)	0.0002
Dysfunctional	1.36	(1.26, 1.47)	< 0.0001
**Time-varying covariates**	**Shift in trajectory in the presence of time-varying covariate**	**95% CI**	**p-value**
Health-related concerns			
High-increasing group	0.65	(−0.27, 1.57)	0.1635
Low-consistent group	1.36	(0.65, 2.07)	0.0001
Difficulty in accessing resources			
High-increasing group	2.02	(1.08, 2.96)	< 0.0001
Low-consistent group	1.48	(0.81, 2.15)	< 0.0001
Caregiving responsibilities			
High-increasing group	1.41	(0.49, 2.33)	0.0028
Low-consistent group	1.21	(0.60, 1.82)	0.0001

^**a**^**Model was adjusted for age, sex, education status, partner status, dwelling type, number of COVID-19 related symptoms, and number of chronic conditions**

## Discussion

This study examined the mental health trajectories of working adults during the COVID-19 pandemic from point of initial lockdown to the gradual lifting of the public health restrictions following the first wave of the COVID-19 pandemic in Hamilton, Ontario. This study identified the pandemic-related stressors and coping strategies associated with depressive symptom trajectories. Our results showed that participants with high depressive symptoms continued to experience an increase in these symptoms even after the easing of the public health restrictions in June 2020. Use of emotion-focused and dysfunctional coping strategies were significant predictors of trajectory group membership. The COVID-19 pandemic-related stressors and experiences also had a significant influence on their respective depressive symptom trajectories.

Our results showed two distinct trajectories with a substantial one third of participants displaying high and increasing depressive symptom patterns. In comparison, a population-based, national survey conducted in the UK during the same time period identified five distinct mental health trajectories [27]. A relatively smaller sample size, a specific target population of employed adults, and differences in the timing of the implementation and lifting of the public health restrictions in our study may explain the different number of trajectories identified between the two studies.

Challenges with accessing resources, including loss of financial resources, and inability to access necessary supplies or food, healthcare, and technology, was significantly associated with depressive symptoms in both groups. Much of the evidence has focused on the impact of job insecurity, unemployment, and loss of income on the risk of anxiety and other mood disorders [28–30]. The results of the current study highlight the negative impact of the pandemic and public health measures on the mental health among individuals who are employed. When public health measures were implemented during the pandemic, many individuals reported being unable to access a range of healthcare supports including regular medical and dental services and testing, physiotherapy, massage therapy, chiropractic services, and counselling services [31]. Lack of access to these services may have led to a worsening of depressive symptoms. In fact, our results demonstrated that experiencing health-related concerns personally or living with a family member who was ill or hospitalized was a significant predictor of depression in the low-consistent group. In addition to reduced access to healthcare services, individuals who were ill or hospitalized for non-COVID-19 related health conditions may also have a fear of acquiring COVID-19, which may further contribute to stress and anxiety [32, 33]. Behavioural activation is one the core approaches for addressing depression. This approach focuses on engagement in activities that bring pleasure and mastery, as opposed to those that can lead to further depression via withdrawal (e.g., social isolation) [34]. Challenges introduced by the pandemic and the lack of access to social supports may make it difficult for individuals who experience depressive symptoms to engage in activities that can help them feel better.

The more time spent in caregiving responsibilities, including caring for young children, adolescents, and older adults as well as problems with providing such care due to pandemic-related restrictions, was a significant predictor of depressive symptoms during the initial months of the COVID-19 pandemic (April–November 2020) in both the low-consistent and high-increasing trajectory groups. Those who worked remotely and/or onsite both experienced a change in their environment, with many having to establish a new work-life routine. Working individuals have sometimes had to balance their work schedules around the needs of other family members and household chores [35]. Research has shown that many parents have experienced an increase in caregiving roles and responsibilities due to childcare and school closures during the lockdown [11]. Further, informal carers providing support to older family members are vulnerable to the consequences of the COVID-19 pandemic. They have reported mental exhaustion and increased stress levels as a result of the uncertainty of the pandemic, and the social distancing measures [36, 37]. Further, informal carers providing emotional support to family members, worrying that their older family member may acquire the infection or that they may transmit it to them, and the difficulties in meeting needs of the older adults during the pandemic have also contributed to higher mental exhaustion and stress [36, 37]. As individuals spend more time in providing support, they may have needed to alter their work or sleep schedules to concentrate on work, which in turn, may contribute to feelings of emotional exhaustion [38]. Evidence also indicates that remote working from home may be more challenging for women as they are often responsible for performing household chores, and taking care of children and supporting them with online schooling [35].

Our results showed that negative coping strategies were associated with higher and increasing levels of depressive symptoms during the COVID-19 pandemic. Compared to the low-consistent depressive symptom trajectory group, membership in the high-increasing depressive symptom trajectory group was associated with higher dysfunctional coping strategies and lower emotion-focused coping strategies. Dysfunctional coping strategies such as denial, venting, substance use, self-blame, behavioural disengagement, and self distraction have been previously shown to be significantly associated with depression, anxiety, and stress [39–41]. Research suggests that dysfunctional coping strategies may be effective in adapting to a stressor in the short-term but are ineffective over longer periods, and may lead to higher levels of stress and depressive symptoms over time [39–41]. On the contrary, positive, emotion-focused strategies have been shown to be an effective coping mechanism. Use of positive reframing, acceptance, and humour to deal with stressful situations and negative emotions in general and during the pandemic have been associated with lower stress and anxiety, and better mental health outcomes [39–41]. However, consistent with other emerging data from the COVID-19 pandemic [39], our results showed that problem-focused coping strategies such as active coping, planning, and seeking instrumental support did not predict depressive symptom trajectories. Although problem-focused coping strategies are known to be associated with mental wellbeing, individuals may seek relatively easier solutions, for example, excessive alcohol consumption, to manage stressors and problematic thoughts [39].

These results could help inform approaches to assist employed individuals working remotely during the COVID-19 pandemic. Managing the COVID-19 pandemic-related stressors and challenges requires a multidimensional approach including innovative strategies (e.g. telehealth) that provide continued access to health care while maintaining public health guidelines, unemployment insurance, access to basic needs and resources, and supportive workplace environment and policies. Employers can also develop programs that raise awareness and educate employees about effective coping skills to help manage depressive symptoms. Recommendations could include encouraging people to seek emotional support from family, friends, colleagues, or counsellors, which may help with depressive symptoms and improve mental health. In this regard, the workplace in our study has demonstrated commitment to promoting the mental health and well-being of all employees and have developed resources and implemented supports including wellness events, additional days off work in the Fall of 2020, and offered extended benefits of an Employee and Family Assistance Program. Moreover, individual departments within the University established wellness groups, developed resources for the department’s website, and offered weekly online drop-in support group to support faculty, staff, and trainees [42]. Similar approaches may also be adopted by other institutions.

### Limitations

The present study has several strengths including the timing of data collection relative to the lockdown restrictions implemented in Ontario, Canada, and use of a longitudinal design to examine depressive symptom trajectories, and to identify factors associated with high and increasing depressive symptoms trajectory among working adults. However, the results of this study should be interpreted considering some limitations. First, our study had a high attrition rate over the follow-up period. Under the missing at random assumption, the PROC TRAJ procedure in SAS allows patterns with incomplete data to borrow information from patterns with more data points and thus participants with missing longitudinal data and time-varying covariates were not excluded from the analysis [26]. However, a greater proportion of individuals who had missing data for depression score had an education attainment below bachelor’s degree and were not married or in a common-law relationship compared to those who had complete data for depression score. Thus, it is possible that the shape and the number of latent trajectories identified may be influenced by the unobserved outcome of those with missing data. Second, our study lacks a pre-pandemic assessment of mental heath from this sample. Nevertheless, our results provide important information regarding the mental health of employed individuals during the initial lockdown of the COVID-19 pandemic and gradual easing of the public health restrictions. Third, our study sample included employed individuals at a single institution and had a relatively higher education attainment, thus the change in depressive symptoms observed in our study may not reflect the change in the general population. Our sample is more representative of the target population but includes a greater proportion of females than the target population. While 22–35% of our sample experienced health-related stressors, difficulties in accessing resources, and increased caregiving responsibilities, caution is warranted when generalizing the findings of this study to other segments of the population. Nevertheless, working at a university is in many ways working for other large institutional employer, as opposed to working for or owning a small business for example, and therefore, similar findings would be expected in other sectors.

## Conclusions

Overall, the results of this study showed that more than one third of working adults at a large institutional employer displayed a pattern of high depressive symptoms that increased over time. COVID-19 pandemic-related stressors and experiences associated with depressive symptom trajectories included health-related concerns, financial challenges and difficulties with accessing resources, and caregiving responsibilities. Further, frequent use of dysfunctional coping strategies and less frequent use of emotion-focused coping strategies were significantly associated with the high and increasing depressive symptom group. The negative mental health impacts of the COVID-19 pandemic and related stressors may persist even after the number of infections drop and restrictions are lifted. Therefore, it is essential to promote emotion-focused strategies and programs that develop awareness, alleviate the depressive symptoms, and promote mental health of working adults.

## Data Availability

The datasets used and/or analysed during the current study are available from the corresponding author on reasonable request.

## Abbreviations

SARS-CoV-2: Severe acute respiratory syndrome coronavirus-2
CESD-10: Center for Epidemiologic Studies Short Depression Scale
AIC: Akaike’s Information Criterion
BIC: Bayesian Information Criterion
OR: Odds ratio
95% CI: 95% confidence intervals
